# Honouring Wishes of Patients: An Islamic View on the Implementation of the Advance Medical Directive in Malaysia

**DOI:** 10.21315/mjms2021.28.2.3

**Published:** 2021-04-21

**Authors:** Mohammad Mustaqim Malek, Shaikh Mohd Saifuddeen, Noor Naemah Abdul Rahman, Aimi Nadia Mohd Yusof, Wan Roslili Abdul Majid

**Affiliations:** 1 Institute of Islamic Understanding Malaysia, Kuala Lumpur, Malaysia; 2 Academy of Islamic Studies, University of Malaya, Kuala Lumpur, Malaysia; 3 Faculty of Medicine, Universiti Teknologi MARA, Shah Alam, Selangor, Malaysia

**Keywords:** end-of-life care, advance medical directive, autonomy, Islamic perspective, euthanasia

## Abstract

An Advance Medical Directive (AMD) is a document in which competent patients express their wishes regarding their preferred choice of future medical plans in the event they become incompetent. AMD is important in relation to the patient’s right to refuse treatment. However, they must also consider cultural and religious values of different communities. In Islam, there are several concerns that need to be addressed, namely the validity of the AMD according to Islamic jurisprudence and patients’ right to end-of-life decision-making. To address these concerns, this article refers to multiple sources of Islamic jurisprudence, such as the *Quran*, the tradition of Prophet Muhammad and the works of Islamic scholars related to this topic. Based on the findings, Islam does not forbid the use of AMD as a method to honour patients’ wishes in their end-of-life care. Islamic jurisprudence emphasises on the importance of seeking patients’ consent before carrying out any medical procedures. However, several conditions need to be given due attention, such as: i) a patient’s cognitive capacity during the process of drawing up an AMD; ii) the professional views of medical experts; iii) the involvement of family members in end-of-life care and iv) the limitations of a patient’s decision-making in creating an AMD.

## Introduction

With progress in the medical field, the quality of healthcare services has improved. Many advanced technologies in the form of new surgical techniques, medical devices and medicines have been developed for healthcare. In end-of-life care, the utilisation of these technologies has contributed towards life sustainability. As a consequence, expectations about medical practitioners’ ability to save lives continue to rise, putting inappropriate pressure on medical practitioners to save every terminally ill patient. Medical practitioners often face a dilemma pertaining to these expectations, especially when the life-sustaining treatment becomes futile.

Thus, there is a need to help medical practitioners decide whether administering life-sustaining treatment is necessary. One method thought to be a possible tool is the Advance Medical Directive (AMD), which is a legislative framework implemented in various jurisdictions around the world (1). An AMD records patients’ medical preferences in advance in case they can no longer make competent decisions about their future treatment choices or preferences (2). The introduction of AMD in decision-making raises issues in medicine, law and ethics (3), which has led to lengthy discussions on the acceptability of AMD in many developed countries, especially in terms of policy development (4).

In Muslim countries, however, AMD is still a new frontier. For Malaysia, in particular, efforts to discuss the issues of AMD and explore their suitability for implementation are very much required, since they are still a novel concept ‘due to the cultural conditions and lack of exposure on the subject matter’ (5). More importantly, the perspective of Islam is pertinent in providing guidelines to the implementation of AMD in Malaysia, since it is a Muslim-majority country. Malaysian Muslims follow the Shāfi‘i school, which is one of the four schools of Islamic law under Sunni Islam. As such, the focus of this article will primarily be on the views of the Shāfi‘i school as they are relevant to the Malaysian context.

Many important issues arise when discussing AMD and this article aims to address two main issues. The first is the validity of AMD according to Islamic jurisprudence and the second is the Islamic view on patients’ right to end-of-life decision-making. In order to discuss the validity of AMD and patients’ right to end-of-life decision-making from the perspective of Islam, this article looks at three major aspects.

The first is to understand the background of AMD, especially from the standpoint of the historical reasons for their emergence and, consequently, the issues that have arisen from them. Since there are established sources in Islam related to the emerging issues vis-à-vis AMD, the second aspect attempts to link these Islamic sources to AMD issues (6). The main sources of Islamic jurisprudence that are used as reference include Islamic juridical sources, namely the *Quran* and Prophetic traditions, as well as contemporary Islamic rulings from *fiqh* councils in various countries. In the Malaysian context, the *fiqh* school of thought referred to is the Shāfi‘i school. After the link between the Islamic sources and AMD issues is established, the third aspect focuses on the analysis of each argument within those sources. If and when AMD is implemented in Malaysia, this research will help to suggest the most suitable and applicable Islamic view on AMD, especially regarding their validity, the scope of patients’ autonomy, the assessment of patients’ competency in decision-making and the limitations on patient’s decision-making, if and when AMD is implemented in Malaysia.

## Understanding the Background of AMD in Healthcare

Dilemmas pertaining to end-of-life decision-making are commonly faced by medical practitioners. Disagreements between family members and medical practitioners regarding the use of life-sustaining treatment occur frequently. Several notable cases are often cited when discussing the complexity of end-of-life situations. For example, in 1975, in the United States of America (USA), a woman named Karen Ann Quinlan was hospitalised in the condition of persistent vegetative state for ten years without any prospect of recovering (7). Quinlan’s adoptive parents and the doctors had conflicting opinions regarding her care. Her adoptive parents believed that the life-sustaining treatment should be withdrawn to allow their daughter to die peacefully. The doctors, in contrast, opposed the adoptive parents’ opinion, saying that withdrawing life support conflicted with their professional judgement. The court found that Quinlan had the right to choose regarding her care, and this right can be transferred to her adoptive parents as her surrogate decision-maker (8).

In 1983, a similar case took place in the USA, involving a woman named Nancy Cruzan. She was in a persistent vegetative state due to the severe injuries she suffered in a car accident. Although she could breathe on her own, she needed help from the feeding tube to receive nutrients and water. Cruzan’s parents thought that their daughter should be allowed to ‘go in peace’ (9). In their opinion, the feeding tube only served to delay the passing of Cruzan. According to them, if it were withdrawn, she would be able to go and rest peacefully. However, the medical practitioners refused to withdraw the feeding tube. Thus, Cruzan’s parents went to court. After seven years of legal trial, in 1990, the Supreme Court found that Cruzan did not want to survive by relying continuously on life aid (10). The feeding tube was withdrawn and Cruzan died a few days later.

These two cases represent the complexity of decision-making process in end-of-life decisions. The Cruzan case was an important benchmark for the passing of the Patient Self-Determination 1990 Act and proxy law in the USA (11). Accordingly, patients are now given the autonomous right to decide their future healthcare as well as to appoint a proxy to decide on their behalf.

In the present context in many developed countries, rights for patients to make decisions pertaining to their end-of-life care are being implemented in the form of AMD. In Asia, the implementation of AMD began to receive attention when Singapore’s former prime minister, Lee Kuan Yew, expressed his end-of-life wishes through an AMD (12). In his last few years, Lee Kuan Yew faced a series of serious illnesses. In 2015, he was rushed to the Singapore General Hospital due to severe pneumonia. He had to rely on life support in the intensive care unit. By then, it was already known that Lee Kuan Yew had stated in his AMD document that if he loses consciousness and recovery is seen as unlikely, he would refuse to continue living by depending on life support. He preferred to be allowed to die naturally without invasive intervention.

Since Kuan Yew’s case, in Malaysia, the local press started covering the importance of AMD in healthcare. The Malaysian Consultative Council of Buddhism, Christianity, Hinduism, Sikhism and Taoism (MCCBHST) also expressed their support towards the implementation of AMD (13). As a religion that emphasises good ethical and moral conduct in every aspect of life, Islam’s stance on AMD need to be resolved; this would prepare the Muslim community if AMD is to be implemented in the future.

## The Validity of AMD according to Islamic Jurisprudence

AMD is a legal document which governs future healthcare preferences and can inform both healthcare professionals and family members of the wishes of the patients as well as their preferred type of care in the event that they cannot express their choices themselves (14). This document helps medical practitioners to decide whether to continue or forego certain medical interventions and allow death to take its natural course.

Besides the term AMD, another common term used to describe this document is ‘living will’, which is a document that is written by a person prior to their death. It contains instructions pertaining to inheritance, guardianship and burial issues. In Islam, a will (*waṣiyyah*) becomes effective after a person dies (15). In contrast, an AMD is effective even before a person dies. Therefore, from the perspective of Islam, the term ‘living will’ is not appropriate when discussing a patient’s preferences in an end-of-life situation.

Discussions concerning AMD in the Islamic framework most suitably fall under the category of medical consent (*idhn al-ṭibb*). Consent (*idhn*) in Islamic jurisprudence is defined as ‘permission that is granted to someone to carry out an action that was initially forbidden’ (16). This definition means that a medical practitioner is originally forbidden to perform medical procedures until he is given permission to do so by patients. Islam emphasises the necessity of obtaining patients’ approval before providing any medical procedures. Furthermore, from the medicolegal perspective, failure to obtain a patient’s consent will result in doctors becoming liable for medical negligence.

AMD is one of the methods that empower patients to choose the type of treatment that they prefer or to refuse treatment being administered to them. A documented AMD has an extended effect, which can represent patients’ wishes in the event they lose the ability to make decisions. The attending doctor is responsible for respecting these wishes.

In Islam, respecting the wishes of patients is in line with the tradition of Prophet Muḥammad which was narrated by his wife, ‘Ā’ishah. This ḥ*adīth* is translated as follows:

*We poured medicine in his (the Prophet’s) mouth but he indicated not to do so. We said, ‘He dislikes the medicine as a patient usually does’. But when he became awake he said, ‘Did I not forbid you from putting medicine (by force) into my mouth? All of you will be forced to take medicine into the mouth, except al-‘Abbās, for he has not witnessed your deed’*. (17)

The consent of a patient to accept or refuse medical treatment corresponds to the above tradition, which was exemplified by Prophet Muḥammad. His refusal of treatment in the above tradition is similar to what is intended by AMD, which is to empower patients to decide and determine their choice of future healthcare. Therefore, it can be inferred from the Prophetic tradition that Islam does not prohibit the implementation of AMD in decision-making.

## Patients’ Autonomy in Islam

The practice of medical paternalism is a topic of much debate in the field of medical ethics (18). At the same time, more and more importance are given to the moral and political freedom of an individual (19). As a result of this development, individual rights have influenced the restructuring of the relationship between doctors and patients in medicine, shifting away from medical paternalism to patient-centred care. Provision of healthcare services has given greater emphasis to respecting patients’ rights to decision-making and to obtaining informed consent before treatment is provided. Respecting patients’ autonomy is to ‘acknowledge their right to hold views, to make choices, and to take actions based on their personal values and beliefs’ (20).

Respecting patients’ wishes regarding how they want to be cared for at the end of their life becomes the main focus of an AMD, and it is designed so that patients can express their wishes and that those wishes will be respected even after they have lost their capacity to decide due to cognitive impairment. Specifically, AMD allows medical practitioners to respect patients’ preferences, including their refusal of medical intervention, honour patients’ rights to their health affairs, and preserve patients’ religious beliefs and dignity.

Regarding patients’ autonomy, Islam does not prohibit anyone to hold views, make choices and take actions based on their personal values and beliefs. These actions are made prior to the full realisation of the situation and understanding on the consequences that may follow. A Prophetic tradition, narrated by ‘Aṭā’ Abῑ Rabāḥ, portrayed a scenario in which a lady agrees to one of the choices proposed by the Prophet, which is translated as follows:

*This black lady came to the Prophet and said, ‘I get epilepsy and my body becomes uncovered, please invoke Allah for me’. The Prophet said to her, ‘If you wish, be patient and you will enter Paradise, and if you wish, I will invoke Allah to cure you’. She said, ‘I will remain patient’, and added, ‘But I become uncovered whenever I get epilepsy so please invoke Allah for me that I may not become uncovered’. So he invoked Allah for her*. (21)

Based on this Prophetic tradition, patients are not prohibited from seeking treatment to cure their illnesses. At the same time, they can also choose to be patient with the illness and abandon seeking treatment. According to al-Ghazzālῑ in his notable work *Iḥyā’ ‘Ulūmuddῑn*, refusing treatment is made due to certain conditions (22), which include the following:

- A person who is suffering from a chronic disease and the recovery is unlikely.
- A person who is terminally ill and death is imminent.
- A person who chooses to stay patient with the hope that the illness that he/she is currently suffering from will elevate his/her status in the sight of God.
- A person who was sinful throughout his/her life chooses to stay patient with the illness so that his/her patience will be the source of forgiveness from God.

Regarding these conditions, the discussion on the moral status of seeking and abandoning medical treatment within the framework of Islamic jurisprudence is required for further deliberation. According to the juridical opinions from the schools of Ḥanafῑ, Mālikῑ, and Shāfi‘ῑ, abandoning treatment is permissible and not considered sinful when there is lack of established efficacy with regards to the treatment (23). Consequently, if there is no treatment with a probable clinical efficacy to cure the illness, especially in the case of the first two conditions that were mentioned by al-Ghazzālῑ, seeking treatment is deemed not to be needed.

In end-of-life cases, the doubtful efficacy of life support is often raised. Based on the juridical argument in the preceding paragraph, end-of-life patients are included among those who are permitted to refuse treatments. Medical practitioners are also not obliged to perform treatments that bring doubtful benefits to the patients (24), particularly life sustaining treatment in end-of-life care. Regarding this matter, the following Islamic ruling from the Kingdom of Jordan is applicable (25):

*… there is no prohibition in Islam to refrain from putting a cancer patient on life support or respirator or dialysis if the medical and treatment team have confirmed and are certain that there is no hope of benefit for the patient in these measures, on the condition that this report is prepared by a medical team consisting of not less than three physicians, being specialists, fair, and trustworthy*.

Furthermore, according to the same Jordanian ruling:

*The patient himself has the right to abstain from treatment if he is content with what Allah has decreed for him (namely, death), and prefers patience to disease*.

This ruling is also in line with the ruling from the Islamic Religious Council of Singapore (26), which states the following:

*It is permissible by Islamic law for a mentally competent individual to make a pledge to refuse the life support treatment in the event of dire straits (terminally ill). It can be assumed that he or she decides to be patient and more willing to die naturally believing that death cannot be avoided at a certain point*.

Islam recognises the patients’ view on how they want to be treated, and this right must always be respected. However, this does not imply that a patient is the sole decision-maker in healthcare. The patients’ autonomy is empowered to liberate patients from external influences in their healthcare management. However, this should not completely eliminate the role of medical professionals and the patients’ family members.

Islam acknowledges the value of experts, especially in highly specialised fields. Experts who are proficient and experienced in their fields of knowledge should always be referred to in order to avoid errors in judgement and decision-making. This view is mentioned in verse 7 of *Sūrah al-Anbiyā’* in the *Quran*:

*So ask the people of knowledge if you do not know*.

Regarding family support, it is important to note that in some cultures, the family unit is very strong. Family members may want to be involved in the decision-making process, including in healthcare. Asian families tend to be involved in healthcare decisions involving their members (27).

In Malaysia, patients appear to be more inclined towards family members’ involvement in the decision-making process (28). Similarly, other Asian countries emphasise family involvement in making medical decisions, as families believe patients may not be able to accept bad news (29). Hence, the role of family members in making decisions for AMD is an important aspect to be taken into consideration in Malaysia. A study on advance directives among the elderly population in Malaysia found that the participants placed more emphasis on the opinion of their family members and doctors than their own. These factors may influence patients’ decisions concerning AMD in Malaysia (2).

## Assessing Patients’ Competency in Decision-Making

In the field of medicine, a doctor is responsible for explaining a patient’s health condition and the treatments available before a patient undergoes any of them. The doctor must achieve patients’ consent and find out their preferences vis-à-vis the treatments options available to them.

For AMD, it is particularly important for medical practitioners to make sure that patients understand the consequences of the decisions they make. AMD primarily deals with the decision to withhold or withdraw treatments, including those that sustain the life of patients. Patients must be able to understand and accept the consequences should life-sustaining therapy be withheld or withdrawn. The attending medical practitioners must be able to identify the capacity of a patient to make decisions. In Malaysia, there is no specific legal provision available to assess mental capacity in general. However, specifically for patients with mental illness, the Mental Health Act 2001 applies. Section 77 of the Mental Health Act 2001 highlights several criteria for assessing patients’ capability to give consent, which are as follows (30):

- Patient understands the condition for which the treatment is proposed
- Patient understands the nature and purpose of the treatment
- Patient understands the risks involved in undergoing the treatment
- Patient understands the risks involved in not undergoing the treatment

In Islamic jurisprudence, the capacity to decide is as important as in conventional law. According to the principle of Islamic jurisprudence *(uṣūl al-fiqh*), the cognitive ability of a person is the foundation of their capacity to decide and to act (*ahliyyah al-adā’*) (31). This principle is based on the basis that a competent and intelligent human being is able to engage in endeavours that fulfil their interest and protect them from harm. A person is considered to be legally competent as long as there are no hindrances or limitations to their intelligence. Hindrances highlighted in the principle of Islamic jurisprudence include madness, dementia, intoxication and folly (32). These hindrances damage the competence of a person in making decisions and giving consent. If consent is taken from a person who suffers from any of these hindrances, it is regarded as invalid.

In the case of Abdul Razak bin Datuk Abu Samah versus Raja Badrul Hisham bin Raja Zezeman Shah & Ors [2013] 10 MLJ 34, the court held that ‘the consent of the spouse may be required when it is evident that the patient is dependent on the spouse to make decisions in regards to the proposed medical treatment or when it is evident to the doctor that the decisions are being made jointly by both spouses in respect of the treatment for one of the spouses’. In this case, the patient, the deceased wife of Abdul Razak (the plaintiff), suffered from adhesion colic and had to undergo an urgent surgery. The patient declined the use of a nasogastric tube before induction of anaesthesia because of the discomfort. Unfortunately, due to this, she developed aspiration pneumonia and died the next day.

The court considered the evidence and concluded that although the deceased was of sound mind, she was very dependent on the plaintiff’s advice in decision-making throughout their married life. In this case, consent was not obtained from the plaintiff. The plaintiff affirmed that if he had known of the lethal risk, he would have persuaded the deceased to use the nasogastric tube. The surgeon was found guilty because he failed to obtain consent from the patient’s husband, which resulted in the patient’s death. The court found that some people are not capable of making decisions on their own due to certain shortcomings, such as misunderstandings or, in the worst case, cognitive impairment. For some people, even if they are mentally capable, they still need support and advice from their family to make decisions. In the abovementioned case, the surgeon had neglected the fact that the patient was incapable of making decisions alone and was dependent on her husband in decision-making throughout her life. The consultation was seen to be inadequate when the husband was not involved in the decision-making process. Hence, it is important to understand patients’ preferences in decision-making and whether to include family members in the process or not.

Adequate time is required to make decisions for an AMD and will involve the assessment of the patients’ cognitive condition to determine their capacity to self-determination. Moreover, end-of-life patients have a higher risk of developing delirium due to the intense distress caused by their illnesses (33). Thus, patients need to be of sound mind during the AMD decision-making process. The involvement of family and relatives is highly encouraged, either to support or to decide on behalf of the patients (in the case of irreversible cognitive impairment, such as dementia).

## Limitations on Patient’s Decision-Making in AMD

Based on the source of Islamic jurisprudence and its principles, Islam holds no prohibition for patients to express their wishes through an AMD. Islam grants permission for a patient to refuse life-sustaining treatment in the event that it gives no benefit and further intervention may cause more physical harm. Medical practitioners in charge must honour the wish of the patients to refuse intervention. However, there are clear limits that must be adhered to in Islam, such as the prohibition of the following: i) euthanasia and assisted suicide; ii) refusal of curative and life-saving treatment and iii) refusal of basic care, which includes provision of artificial nutrition and hydration if the intention is to hasten death.

### Euthanasia and Assisted Suicide

Although it is allowed for a patient to forgo treatments that delay the death process, Islam strictly prohibits the termination of life through euthanasia and assisted suicide (34). This is based on the specific prohibition to take one’s life and commit suicide. The *Quran* declares in verse 29 of *Sūrah al-Nisā’*:

*Do not kill yourselves, for verily God has been to you Most Merciful*.

The primary aims in healthcare services are to provide care and alleviate pain. Euthanasia and assisted suicide hasten the death process to alleviate the prolonged suffering of patients. However, Islam does not recognise these methods to alleviate pain. Both acts violate the fundamental teachings in Islam that emphasises the protection of life (ḥ*ifẓ al-nafs*). A person has no right to take their own life or another person’s life even if it is in the name of mercy. From the perspective of Islam, killing and suicide taint the sanctity of life. Preservation of life is one of the five higher objectives of Islamic law (*maqāṣid al-sharῑ‘ah*), which, in essence, aims to protect the general well-being (*maṣlaḥaḥ*) of mankind (35). Therefore, Islam does not grant permission for patients to dictate medical practitioners to end their life through euthanasia and assisted suicide. An AMD should be utilised to help doctors to make difficult decisions when patients become incompetent to make their own decisions. These decisions, however, should not include the advocacy of euthanasia and assisted suicide.

### Refusal of Curative and Live-Saving Treatment

It is clear from various Islamic sources that withholding and withdrawing treatment is permissible in the case where treatment is futile and there is little hope for recovery. Therefore, AMD allows patients to refuse medical treatment that is no longer effective in treating the illness. However, in the case of curable diseases, in which non-treatment will result in greater harm including death, withholding and withdrawing treatment is not permissible.

Seeking treatment is generally permissible according to the jurists from the school of Shāfi‘ῑ (36). However, this general moral status may change in life-threatening situations. This topic is discussed in one of the chapters in Islamic jurisprudence known as *rukhṣah*, which is a term in Islamic law that provides a concession for Muslims when faced with difficult situations in performing religious duties (37). *Rukhṣah* seeks to eliminate the difficulties faced by individuals or communities by providing facilitative religious positions that enable Muslims to perform their religious precepts and obligations within any limitations or challenges they may face (37).

Avoiding and removing illness are emphasised in Islam to the point of granting *rukhṣah*. In certain cases, choosing *rukhṣah* is obligatory if the original duty may lead to harm (38). For example, it is emphasised by the jurists from the school of Shāfi‘ῑ that if a medical practitioner informs a patient that using water will worsen their illness, the patient is exempted from performing ablution (*wuḍū’*), and this can instead be replaced with dry ablution (*tayyammum*) (39). In this example, treatment is obligatory for a person who suffers from a wound that can lead to a greater harm. Similarly, in the case of fasting in the month of Ramaḍān, if a patient is required to take medication to cure their illness, and thus avoiding fatal harm, then they are required to break their fast to take the medication. It is imperative that doctors treating the patient provide adequate information to the patient so that the patient understands the importance of treatment in saving their life (40). Using similar reasoning, taking preventive health measures to avoid the likelihood of harm towards the person is obligatory (23). Other examples include receiving vaccinations to prevent the spread of infectious diseases or taking medications for mental problems to avoid harming other people.

The preservation of life is one of the main objectives of Islamic law. If the available efforts of treatment are effective to treat illnesses, thus avoiding greater harm, such as permanent disability and death, it is obligatory for a patient to take the treatment (41). Another major concern regarding medical treatment in Islam revolves around the certainty of clinical efficacy to cure illnesses in which the goal is to prevent harm (23). The criterion, ‘certainty,’ is based on the assessment made by medical practitioners which is derived from established medical knowledge and clinical practice that the treatment can remove illness-related harms, including death (39). This criterion sets the threshold where the clinical point of view is prioritised over the patient’s personal choice. Therefore, refusing effective treatment, in this instance, is not permissible, and it shall not be included as one of the contents in AMD.

### Provision of Artificial Nutrition and Hydration

An AMD is not applicable when a patient decides to refuse basic care, which includes warmth, the necessities to maintain personal cleanliness as well as the provision of food, water and shelter (42). However, the offer of food and water does not include artificial nutrition and hydration (42), as these are considered a medical treatment and should be treated as any other medical intervention (43). They are provided to patients via any form of tube feeding for nutrition and intravenous or subcutaneous infusion of fluids for hydration; this is known as clinically assisted nutrition and hydration (CANH).

Since CANH includes the provision of nutrition and hydration to patients, it can be contentious when the decision to withdraw such treatment is made in the event that doctors believe that the treatment is no longer in the patients’ best interest. Dispute arises because some may argue that the provision of CANH is considered a necessity and should always be provided to patients (44). Therefore, it is important that doctors treating the patients are aware of these views. In Malaysia, one challenge is that there is still no specific guideline available regarding CANH (45). Hence, this issue still poses a dilemma in daily medical practice and is a challenge in the implementation of AMD.

It is clear from the previous discussion that Islam is of the view that in the event a patient is suffering from a terminal illness which will inevitably lead to death, medical treatment, including life support, can be discontinued. However, when CANH is discussed from an Islamic perspective, it should be differentiated from life-support because CANH is considered basic care, and it is similar to regular food and drinks (46). It is an obligation to feed patients who are no longer capable to feed themselves (24). As a result, when CANH is deemed necessary, the act of withholding or withdrawing nutrition would not be appropriate because this would lead to death by starvation and not by the underlying terminal illness (47). Regarding AMD, from an Islamic point of view, when CANH is deemed necessary, the act of refusing it with the intention to die faster should not be included as one of the patients’ decisions in the document.

However, when the provision of CANH brings more harm than good based on the expert opinion of the attending doctors, then withholding or withdrawing CANH should take place in the best interest of the patients. This is because, in many cases, the condition of terminally ill patients can deteriorate due to the complications associated with CANH, such as aspiration pneumonia, dyspnoea, nausea, diarrhoea and hyperkalemia (48). Therefore, the harmful effects of CANH outweigh its benefits, rendering it no longer suitable and the obligation to provide CANH may be lifted (49). From the Islamic perspective, clinical assessment by the attending doctors regarding the extent of CANH provision play a major role in fulfilling the patients’ best interest.

## Conclusion

The AMD is an innovative approach in medical care; they are a response to greater awareness concerning the personal rights and autonomy of patients. AMD serves the purpose of honouring patients’ preferences in healthcare, particularly in end-of-life situations. In the context of Malaysia, AMD is expected to become more important, especially consider the increasing number of palliative care patients, which is projected to steadily rise to 239,173 people by the year 2030 (50). As such, discussions on AMD are critical, especially how they fit into Islamic jurisprudence. Islam as a part of the moral and ethical ecosystem has its own perspective when dealing with emerging challenges in the modern era, including AMD.

The Islamic perspective on AMD primarily revolves around Islamic principles and values. It is pertinent to note that while the Islamic principles and values serve as guidance, they should not be looked at as a blanket ruling. Instead, assessing the content of AMD from the Islamic perspective must be made on a case-by-case basis, with the Islamic principles and values acting as a general guideline. The Islamic principles and values as discussed in this article vis-à-vis Islam and AMD are as follows:

- Islam permits the application of AMD as a way to honour patients’ wishes to accept and refuse treatment in end-of-life settings. A person has the right to decide their preferred type of care through an AMD
- It is allowed in Islam for a person with a sound mind to refuse the use of life-sustaining treatment in the future if they are terminally ill with no hope of recovery and if this choice is verified by medical experts
- It is highly recommended that patients involve their family members and relatives during the process of decision-making for an AMD. This would enable family members and relatives not only to be informed of the patients’ wishes but also to understand the reasoning behind their wishes
- Islam highly regards medical professionals due to their knowledge, proficiency and experience. Therefore, the emphasis on patients’ autonomy should never eradicate the role of experts to actively assist and advise in any decision-making process
- While Islam allows AMD to be utilised by patients in choosing their preferred treatments, Islam prohibits certain choices in order to preserve the sanctity of life. The prohibited choices include euthanasia and assisted suicide, the refusal of curative and life-saving treatment, and the refusal of basic care, which includes the provision of artificial nutrition and hydration with the intention of hastening the death process
- Attending doctors should respect and honour the wishes made by patients in AMD as long as the decisions made are within the Islamic principles and values as discussed

As noted in this article, AMD is still a novel concept in a country like Malaysia. While this article has explored what can be considered as the basics of AMD implementation in a Muslim-majority country like Malaysia, more research on AMD is needed. At the same time, engagement with various stakeholders needs to be done. Equally important is the need to create awareness and provide adequate information on AMD to members of the public so that they may better understand it.
